# The potential role of next-generation sequencing in identifying *MET* amplification and disclosing resistance mechanisms in NSCLC patients with osimertinib resistance

**DOI:** 10.3389/fonc.2024.1470827

**Published:** 2024-10-21

**Authors:** Xiao Xiao, Ren Xu, Jun Lu, Beibei Xin, Chenyang Wang, Kexin Zhu, Hao Zhang, Xinyu Chen

**Affiliations:** ^1^ School of Physics, Changchun University of Science and Technology, Changchun, China; ^2^ Research & Development Department, Shanghai Rightongene Biotechnology Co., Ltd., Shanghai, China; ^3^ Department of Respiratory and Critical Care Medicine, Shanghai Chest Hospital, Shanghai Jiao Tong University School of Medicine, Shanghai, China; ^4^ School of Life Science and Technology, Changchun University of Science and Technology, Changchun, China

**Keywords:** next generation sequencing, non-small cell lung cancer, MET amplification, osimertinib resistance, fish

## Abstract

**Purposes:**

Osimertinib, one of the third-generation *EGFR*-tyrosine kinase inhibitors (TKIs) designed to target *EGFR* T790M mutation, significantly improves the prognosis of lung cancer. However, drug resistance still happens and *MET* amplification is responsible for one of the main causes. Fluorescence *in situ* hybridization (FISH) is the gold standard for *MET* amplification detection, but fundamentally limited by observer subjectivity. Herein, we assessed the value of next-generation sequencing (NGS) method in *MET* amplification detection in non-small cell lung cancer (NSCLC), as well as revealed the mutation profiling of NSCLC patients with osimertinib resistance to provide some valuable clues to the mechanisms of resistance.

**Methods:**

A total of 317 cancer tissue samples from 317 NSCLC patients at time of progression following osimertinib were submitted to NGS and only 96 tissues were tested by FISH simultaneously. With FISH results as gold standard, enumeration algorithm was applied to establish the optimal model for identifying *MET* amplification using gene copy number (GCN) data.

**Results:**

The optimal model for identifying *MET* amplification was constructed based on the GCN of *MET*, *BRAF*, *CDK6* and *CYP3A4*, which achieved a 74.0% overall agreement with FISH and performed well in identifying *MET* amplification except polysomy with a sensitivity of 85.7% and a specificity of 93.9%. The inconsistency between NGS and FISH occurred mainly in polysomy subtype, while *MET* GCN ≥ 5 could be reliably recognized by NGS. Moreover, the most frequently mutated genes in NSCLC patients with osimertinib resistance were *EGFR* (59.94%), followed by *TP53* (43.85%), *NRG1* (9.46%), *PIK3CA* (6.31%), and ATM (5.36%). The known resistance mechanisms, including *MET* amplification, *EGFR* (C797S, L718Q/R), *TP53*, *CDK4*, *CDK6*, *CDKN2A*, *BRAF*, *KRAS*, *NRAS* and *PIK3CA* mutations were also disclosed in our cohort.

**Conclusions:**

NGS assay can achieve a high concordance with FISH in *MET* amplification detection and has advantages in portraying various genetic alterations, which is of worthy in clinical promotion.

## 1 Introduction

Lung cancer is the leading cause of cancer-related mortality worldwide. Non–small cell lung carcinoma (NSCLC) constitutes approximately 85% of all the lung cancers and has a poor 5-year survival rate of ~20% (1, 2), despite great efforts made over the past decades. The development of epidermal growth factor receptor (*EGFR*)-tyrosine kinase inhibitors (TKIs) is an important milestone in the targeted therapy of NSCLC (3). Numerous clinical trials have demonstrated that both the first-generation *EGFR*-TKIs such as gefitinib and erlotinib, and the second-generation *EGFR*-TKIs represented by afatinib achieved superior efficacy in the treatment of the *EGFR*-mutant NSCLC patients (4, 5). However, most NSCLC patients develop drug resistance, with *EGFR* T790M mutation as the most common resistant mechanism (6). To overcome the T790M-mediated resistance, the third-generation *EGFR*-TKIs, osimertinib, targeting the T790M mutation emerged as the times require. However, patients also inevitably develop resistance, which limits the long-term efficacy of third-generation *EGFR*-TKIs in the clinic (7, 8). Of note, osimertinib has been recommended as the preferred first-line treatment option for *EGFR*-mutated NSCLC at present (9). Therefore, it is necessary to comprehensively explore the resistance mechanisms of osimertinib.

Mesenchymal-epithelial transition (*MET*) gene located on chromosome 7 (7q31) encodes the receptor tyrosine kinase or hepatocyte growth factor receptor (HGFR). HGFR, along with its ligand, HGF, functions as an important regulator of cell survival, proliferation, motility and migration (10, 11). Dysregulation of *MET* signaling, such as *MET* amplification, *MET* exon 14 skipping mutation, and *MET* overexpression has been found to be associated with the development of lung cancer (12). *MET* amplification referring to the *MET* gene copy number (GCN) gains occurs in 1-6% of newly diagnosed NSCLC patients and is considered as a poor prognostic marker (13, 14). Moreover, growing evidence has demonstrated that *MET* amplification is a key driver of acquired resistance to *EGFR*-TKIs in addition to *EGFR* mutation, especially for patients resistant to the third-generation of *EGFR*-TKIs (15). Detailly, the incidence of *MET* amplification in NSCLC patients resistant to third-generation *EGFR*-TKIs increased from 5-22% to 5-50% compared to the patients’ resistance to the first/second-generation *EGFR*-TKIs (16). Of note, some clinical studies have demonstrated that the combination therapies of *EGFR*-TKIs and *MET* inhibitors improved the outcomes of NSCLC patients with *MET* amplification (17, 18). Therefore, accurate detection of *MET* amplification is essential for NSCLC patients with *MET* amplification.

To date, various techniques, including fluorescence *in situ* hybridization (FISH), quantitative real-time polymerase chain reaction (qRT-PCR) and next-generation sequencing (NGS), have been developed for detecting *MET* GCN (19). Both polysomy (multiple copies of chromosome 7) and *MET* amplification (multiple copies of *MET* only) cause *MET* GCN gains. FISH is the gold standard for detecting *MET* amplification status, but fundamentally limited by reliance on the subjectivity of the observers (20). NGS is increasingly applied in clinical practice for the detection of *MET* GCN, which offers comprehensive profiling not only *MET* amplification, but also many other genetic alterations that cannot be detected by FISH but are of high clinical significance. To date, several studies have explored the consistency between NGS and FISH for *MET* amplification detection. Unfortunately, the concordance between the FISH and NGS was no more than 70% (21, 22). Also, no consensus on the definition of *MET* amplification is reached, with the cutoff values ranging from GCN 2.3-10 depending on different NGS platforms (16, 22). In addition, *MET* amplification is regarded as a truly oncogenic driver compared to polysomy (23, 24). Therefore, it is necessary to find out a standardized NGS method to effectively define *MET* amplification.

**Figure 1:**
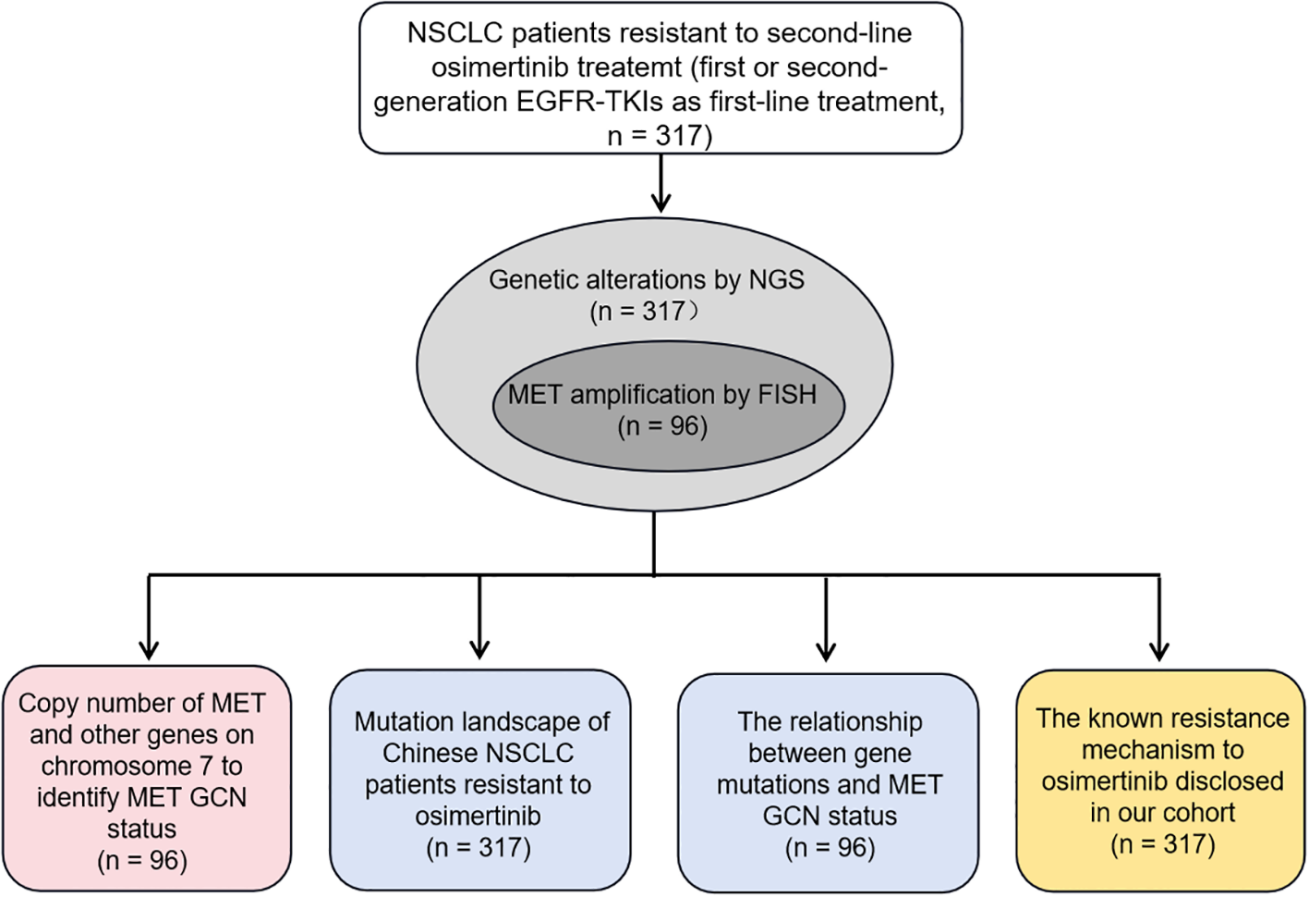
The experimental flowchart of this study.

In our study, we mainly investigated whether NGS could serve as an alternate method to identify *MET* amplification status, especially *MET* amplification, and reported the mutation profiling of the largest cohort of Chinese NSCLC patients with osimertinib resistance to reveal the underlying mechanisms to resistance. To this end, we first constructed the optimal model to identify *MET* amplification based on the GCN of *MET* and other genes located on chromosome 7 in 96 NSCLC patients, with FISH results as the gold standard. Then, we described the genetic mutation profile of 317 patients with resistance to osimertinib, and explored the relationship between genetic mutation and *MET* amplification status. Finally, we disclosed the known mechanisms of resistance to osimertinib in our cohort. **Figure 1** showed the experimental flowchart of this study.

## 2 Method

### 2.1 Patients and samples

A total of 317 NSCLC patients from Shanghai Chest Hospital between August 2021 to October 2022 were enrolled in this study. All cases received first- and/or second-generation *EGFR*-TKIs as the first-line treatment, followed by third-generation *EGFR*-TKIs (osimertinib) as the second-line treatment, and eventually showed drug resistance. The tissue samples from 317 NSCLC patients at the time of progression following osimertinib were collected for genetic abnormality analysis, of which all cases were analyzed by NGS and only 96 by FISH simultaneously. Therefore, 96 NSCLC cases could be directly compared using both NGS and FISH regarding the determination of the *MET* amplification status. Clinic data including the age, gender, stage, and histology type were collected from the medical records. Informed consents were obtained from all patients and this study was carried out in compliance with the Declaration of Helsinki and approved by the ethics committee of Shanghai Chest Hospital (No. KS(Y)22288).

### 2.2 Fluorescent *in situ* hybridization

FISH was performed on 4-μm-thick formalin-fixed, paraffin-embedded (FFPE) tissue sections using a *MET*/*CEP7* (centromere of chromosome 7) dual-color FISH probe (Vysis, Abbott Laboratories, Illinois, USA) according to the manufacturer’s instructions. The mean copy number of *MET* and *CEP7* was recorded in at least 50 non-overlapping tumor cell nuclei and then *MET*/*CEP7* ratio was calculated. *MET* amplification was defined as a mean *MET*/*CEP7* ratio ≥  2, and polysomy was defined as a mean GCN ≥ 5 and *MET*/CEP7 ratio < 2 synchronously (25).

### 2.3 Next-generation sequencing

Genomic DNA (gDNA) was extracted from FFPE tissues using the QIAamp FFPE DNA Tissue Kit (Qiagen, Germantown, MD, USA). In general, 200 ng gDNA was used to create the sequencing library targeting 84 genes (listed in **Supplementary Table 1**) (Rightongene, Shanghai, China) using a custom hybrid-capture NGS panel. Briefly, the DNA was fragmented and the fragmented DNA was subjected to end-repairing, A-tailing, and ligation with indexed adapters. Then, the libraries were PCR-amplified and purified for target enrichment. The library concentration was recorded by Qubit 3.0 Flourometer (Thermo, Massachusetts, USA) and the length and purity of the library fragment were measured by Qsep100 automated nucleic acid protein analysis system (BIOptic, Jiangsu, China). 500 ng indexed DNA libraries were pooled to obtain 1.5 μg DNA. Pooled DNA samples were mixed with DNA blocker and dried in a concentrator. Hybridization Master Mix was added to each sample. The mixtures were incubated at 95 °C for 10 min, then combined with probes and incubated at 65 °C for 16 h. Target regions were captured in accordance with the manufacturer’s instructions. The concentration and fragment size distribution of the final library were determined using Qubit 3.0 fluorometer and Qsep100 automated nucleic acid protein analysis system, respectively. Targeted NGS was performed on the Novaseq platform (Illumina, California, USA). The NGS detection sensitivity was approximately 0.1%.

### 2.4 Bioinformatic analysis

Original sequencing image data file was converted into the sequencing data through base recognition and stored as FASTQ file. The quality of the original sequencing data was evaluated by FastQC (version 1.11.4) software. Trimmatic (version 3.6) software was used to remove joint information, low-quality bases, or undetected bases. After sorting and eliminating repetitive sequences, BAM files were obtained. The data of BAM files including library average length, comparison rate, coverage, capture rate, sequencing depth, homogeneity was used to evaluate the quality of sample library. Based on the BAM results, the SNP and InDel sites were detected by GATK and Mutet2 (26, 27) and annotated by ANNOVAR (28). Germline variants were removed using the ExAC (29), and 1000 Genomes project (30) (>0.1% population frequency). In addition, mutation meeting the following criteria were filtered out: (1) variant allele frequency (VAF) of mutation was less than 1%; (2) mutations predicted as harmless mutation by SIFT (SIFT score > 0.05) (31), and Polyphen2 (Polyphen2 HDAV score ≤ 0.446) (32). The final retained variants may be deleterious, likely deleterious or unknown significance. For the gene copy number identification, we first established a baseline of relative read coverage for capture region using normal tissue samples and then compared clinical samples to this baseline. Variant calling and copy-number variation analysis were performed by the cnvkit (https://github.com/etal/cnvkit).

### 2.5 Construction of the optimal model to identify *MET* amplification

Enumeration algorithm was applied to construct the optimal model to identify *MET* amplification using GCN data in 96 patients with FISH results. Receiver operating characteristic (ROC) curves were used to identify cutoff values of CN (*MET*)/CN (*EGFR*, *BRAF*, *CDK6*, *PMS2*, *ABCB1* and *CYP3A4*, which were all of other genes on the 84-gene panel that were on chromosome 7) distinguishing between *MET* amplification subtype and polysomy/negative subtype. Then, enumeration algorithm was used to find out the optimal combination of CN (*MET*)/CN (other genes on chromosome 7) with the highest consistency with FISH in identifying *MET* amplification. Finally, the ROC curve was used to determine the cutoff value of *MET* GCN distinguishing between polysomy subtype and negative subtype. The sensitivity and specificity for identifying *MET* amplification were calculated as the proportion of *MET* amplification cases identified by NGS among *MET* amplification cases identified by FISH, and the proportion of polysomy or negative cases identified by NGS among polysomy or negative cases identified by FISH, respectively. The sensitivity and specificity for identifying polysomy or negative cases were calculated in the same way.

### 2.6 Statistical analysis

Statistical analysis was performed using R package (version 4.1.2). Categorical variables were evaluated using the chi-squared test or Fisher’s exact test. All tests were two-tailed, *p* < 0.05 was considered to be significantly different. * represents *p* < 0.05 and ** represents *p* < 0.01. Graphs were made in Prism 8, v8.2.0 (GraphPad Software Inc.) and Adobe Illustrator 2021 (Adobe Inc.).

## 3 Results

### 3.1 Baseline characteristics

A total of 317 NSCLC patients were enrolled in this study, with a median age of 60 years (range from 32 to 84 years). 135 (42.6%) patients were male and most of patients (94.2%) were diagnosed at an advanced stage of IV. 306 (96.8%) cases were lung adenocarcinoma, 9 (2.8%) cases were squamous cell carcinoma, and 1 (0.3%) case was adenosquamous carcinoma. 96 patients were subjected to FISH testing for detecting MET amplification status, where 14 (14.6%) cases were *MET* amplification, 16 (16.7%) cases were polysomy and 66 (68.8%) cases were negative. A summary of the clinical characteristics of 317 NSCLC patients was presented in **Table 1**. There was no significant difference in clinical characteristics between patients with MET amplification and those without MET amplification (**Table 2**).

**Table 1:** Clinical characteristics of 317 patients with NSCLC.

Variables	Number of patients (%)
Age (years)	
Median	60
Range	32-84
Gender	
Male	135 (42.6)
Female	182 (57.4)
Clinical stage	
III	6 (5.8)
IV	97 (94.2)
NA	214
Histology type	
LUAD	306 (96.8)
LUSC	9 (2.8)
ASC	1 (0.3)
NA	1
*MET* amplification status identified by FISH	
*MET* amplification	14 (14.6)
Polysomy	16 (16.7)
Negative	66 (68.8)
NA	221

LUAD, lung adenocarcinoma; LUSC, lung squamous cell carcinoma; ASC, adenosquamous carcinoma.

**Table 2:** Differences in clinical characteristics between patients with *MET* amplification and those without *MET* amplification.

Variables	Patients with *MET* amplification by FISH (n=14)	Patients without *MET* amplification by FISH (n=82)	*P* value
Age (years)			0.848
Median	59.5	59.5	
Range	33-77	41-82	
Gender			1.000
Male	7 (50)	43 (52.4)	
Female	7 (50)	39 (47.6)	
Clinical stage			1.000
III	1 (7.1)	5 (6.1)	
IV	13 (92.9)	77 (93.9)	
Histology type			0.554
LUAD	13 (92.9)	78 (95.1)	
LUSC	1 (7.1)	3 (3.6)	
ASC	0 (0)	1 (1.2)	

### 3.2 The optimal model to identify *MET* amplification by NGS

**Figure 2:**
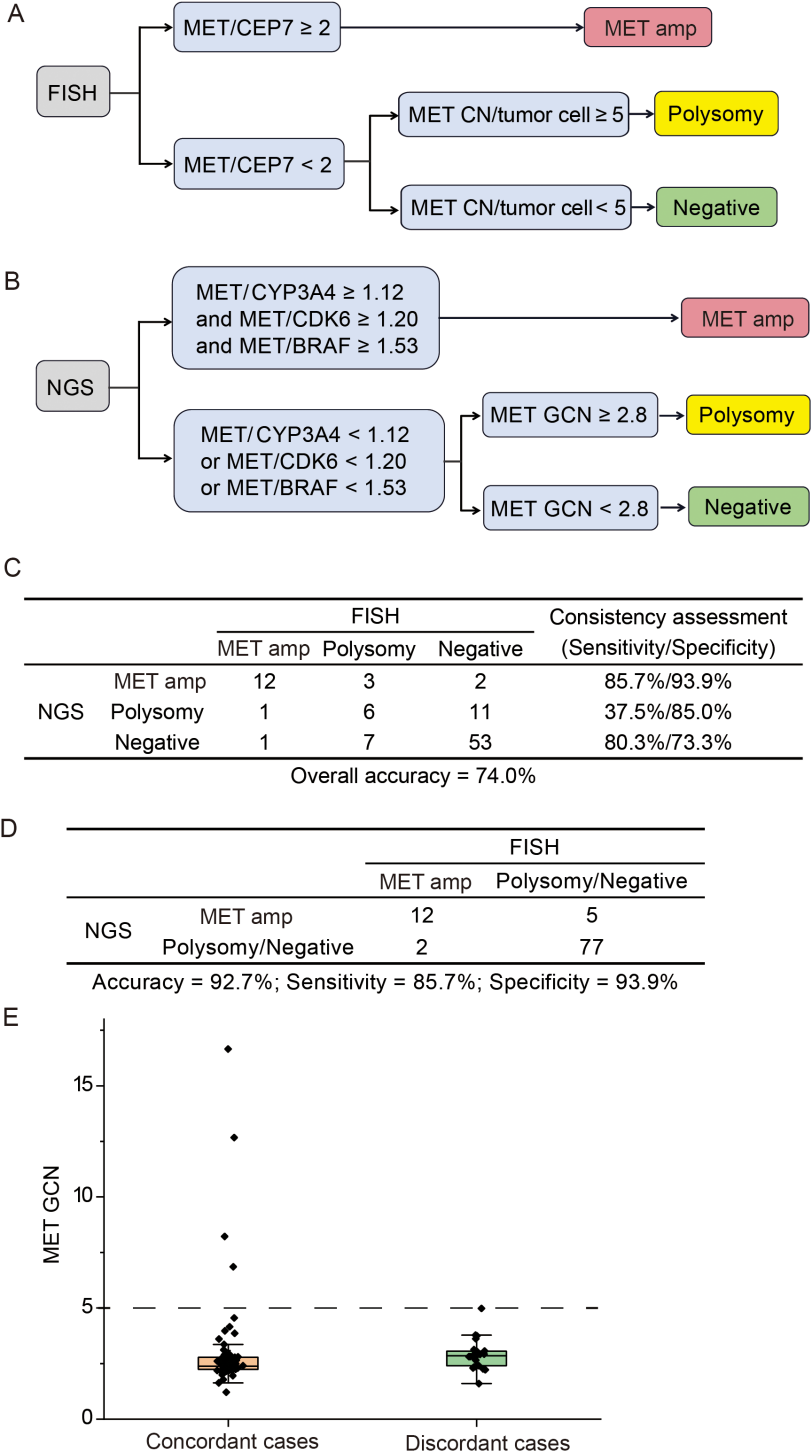
Comparison of NGS and FISH for detecting *MET* gene copy number status. **(A)** The criteria for defining *MET* amplification status via fluorescence *in situ* hybridization (FISH). **(B)** The optimal model for identifying *MET* amplification status via next-generation sequencing (NGS). **(C)** The concordance between NGS and FISH in identifying *MET* amplification, polysomy and negative. **(D)** The consistency between NGS and FISH in identifying *MET* amplification vs. other cases. **(E)** Distribution of *MET* gene copy number for concordant and discordant cases. Amp, amplification; GCN, gene copy number.

The commonly accepted criteria for defining *MET* amplification status via FISH was showed in **Figure 2A**. With FISH results as a reference, we explored the appropriate model to define *MET* amplification status based on the GCN of *MET* and other genes (*EGFR*, *BRAF*, *CDK6*, *PMS2*, *ABCB1* and *CYP3A4*) located on chromosome 7. The optimal model demonstrated that cases with *MET*/*CYP3A4* ≥ 1.12, *MET*/*CDK6* ≥ 1.20 and *MET*/*BRAF* ≥ 1.53 were predicted to be *MET* amplification. Among the other cases, the cases with *MET* GCN ≥ 2.8 were predicted to be polysomy; and cases with *MET* GCN < 2.8 were predicted to be negative (**Figure 2B**). The overall concordance between NGS and FISH was 74.0% (95% CI 64% - 82.4%), while the sensitivity/specificity for identifying *MET* amplification, polysomy and *MET* negative were 85.7%/93.9%, 37.5%/85% and 80.3%/73.3%, respectively (**Figure 2C**). The model was stronger in identifying *MET* amplification and negative with a sensitivity of 85.7% and 80.3%, respectively; and weaker in identifying polysomy with a sensitivity of 37.5%. When combining polysomy and negative into one group, the overall concordance between NGS and FISH reached 93.9% (**Figure 2D**), suggesting that polysomy might be the key factor contributing to the discrepancy between NGS and FISH in the detection of *MET* amplification status.

Next, we investigated whether the concordance between NGS and FISH might be mediated by *MET* GCN levels. The distribution of *MET* GCN in all cases ranged from 1.2 to 16.7. The *MET* GCN of 25 discordant cases between NGS and FISH was predominantly distributed between 2 and 5, and the NGS method showed a 100% concordance with FISH when *MET* GCN was ≥ 5 (**Figure 2E**). Overall, our optimal model performed well in identifying *MET* amplification, especially the high level amplification.

### 3.3 Landscapes of genetic mutations in patients resistant to third generation TKIs

**Figure 3:**
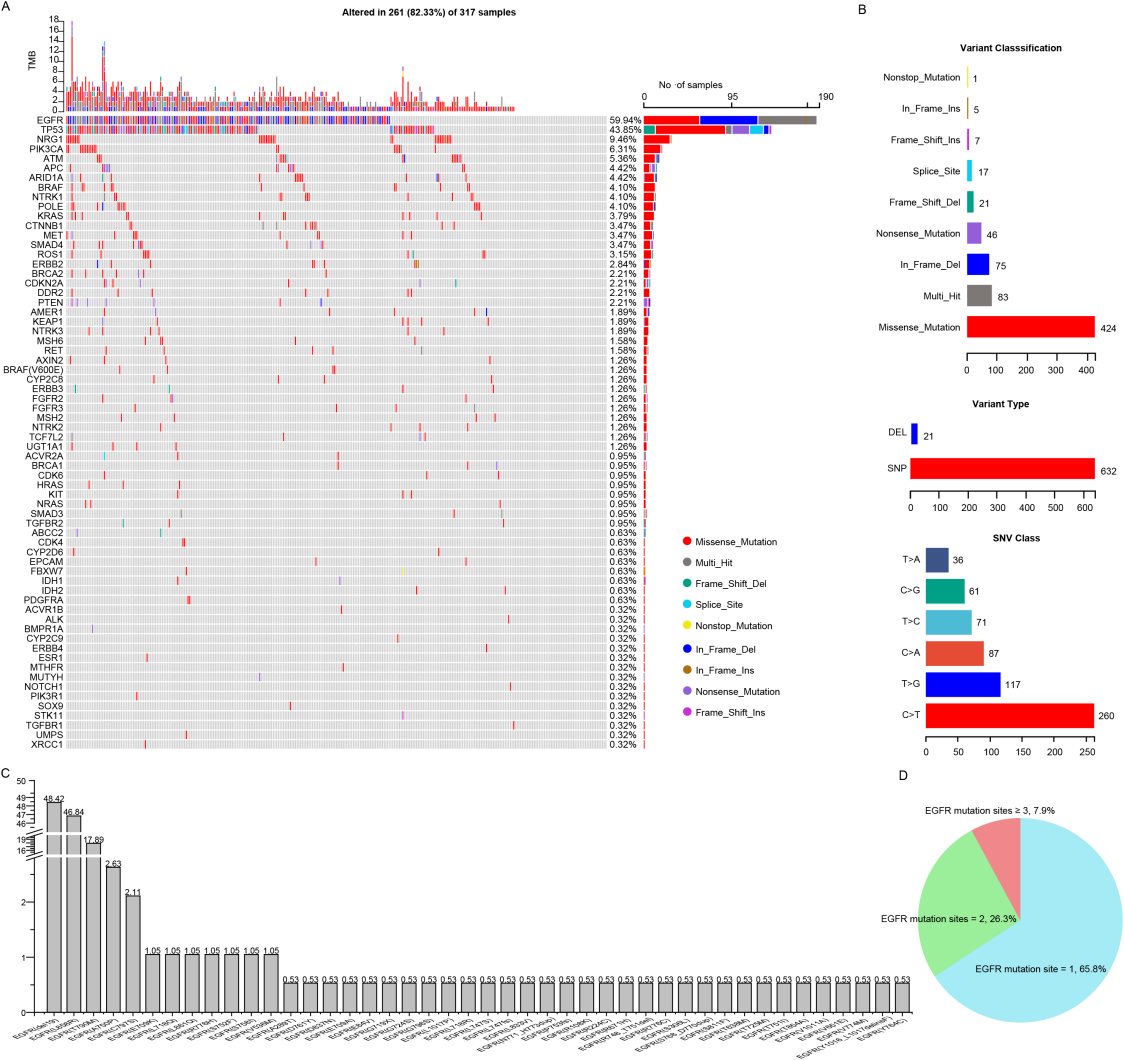
Comprehensive mutation analysis of Chinese NSCLC patients with osimertinib resistance. **(A)** Gene mutational landscapes of NSCLC patients with osimertinib resistance. **(B)** Classifications of mutation forms and mutation types in NSCLC patients with osimertinib resistance. **(C)** Bar chart showing the mutation frequencies of *EGFR* mutation sites. **(D)** Pie chart showing the percentage of patients carrying different numbers of *EGFR* mutation sites.

The targeted sequencing was performed in all the patients and the mutation landscape of NSCLC patients resistant to osimertinib was showed in **Figure 3A**. A total of 66 mutated genes and 488 mutation sites were detected in 82.33% (261 of 317) of the NSCLC patients, among which the most frequently mutated genes were *EGFR* (59.94%), followed by *TP53* (43.85%), *NRG1* (9.46%), *PIK3CA* (6.31%), *ATM* (5.36%), *APC* (4.42%), *ARID1A* (4.42%), *BRAF* (4.10%), *NTRK1* (4.10%), *POLE* (4.10%) and *KRAS* (3.79%). Those mutations had multiple mutation forms (missense, nonsense, nonstop, frameshift/inframe and splicing mutations) and mutation types (C→T, C→A, C→G, T→C, T→A and T→G), where the most commonly mutation form and type were missense mutations (424, 62.%) and C→T transitions (260, 41.1%), respectively (**Figure 3B**). Among the *EGFR* mutant patients, 95.79% of the patients harbored the reported hotspots, including exon 19 deletion (48.42%), L858R (46.84%) and T790M (17.89%), respectively. Moreover, uncommon mutation sites such as A750P (2.63%), C797S (2.11%), E709K (1.05%) accounted for 20.59% of *EGFR* mutations (**Figure 3C**). The majority of patients (65.8%) possessed single *EGFR* mutation site, a decent number of patients (26.3%) had two *EGFR* mutation sites, and the minority of patients (7.9%) had more than two *EGFR* mutation sites (**Figure 3D**). These mutational analyses may provide some valuable clues to the mechanisms of osimertinib resistance.

### 3.4 Correlations between genetic mutations and *MET* amplification status

**Figure 4:**
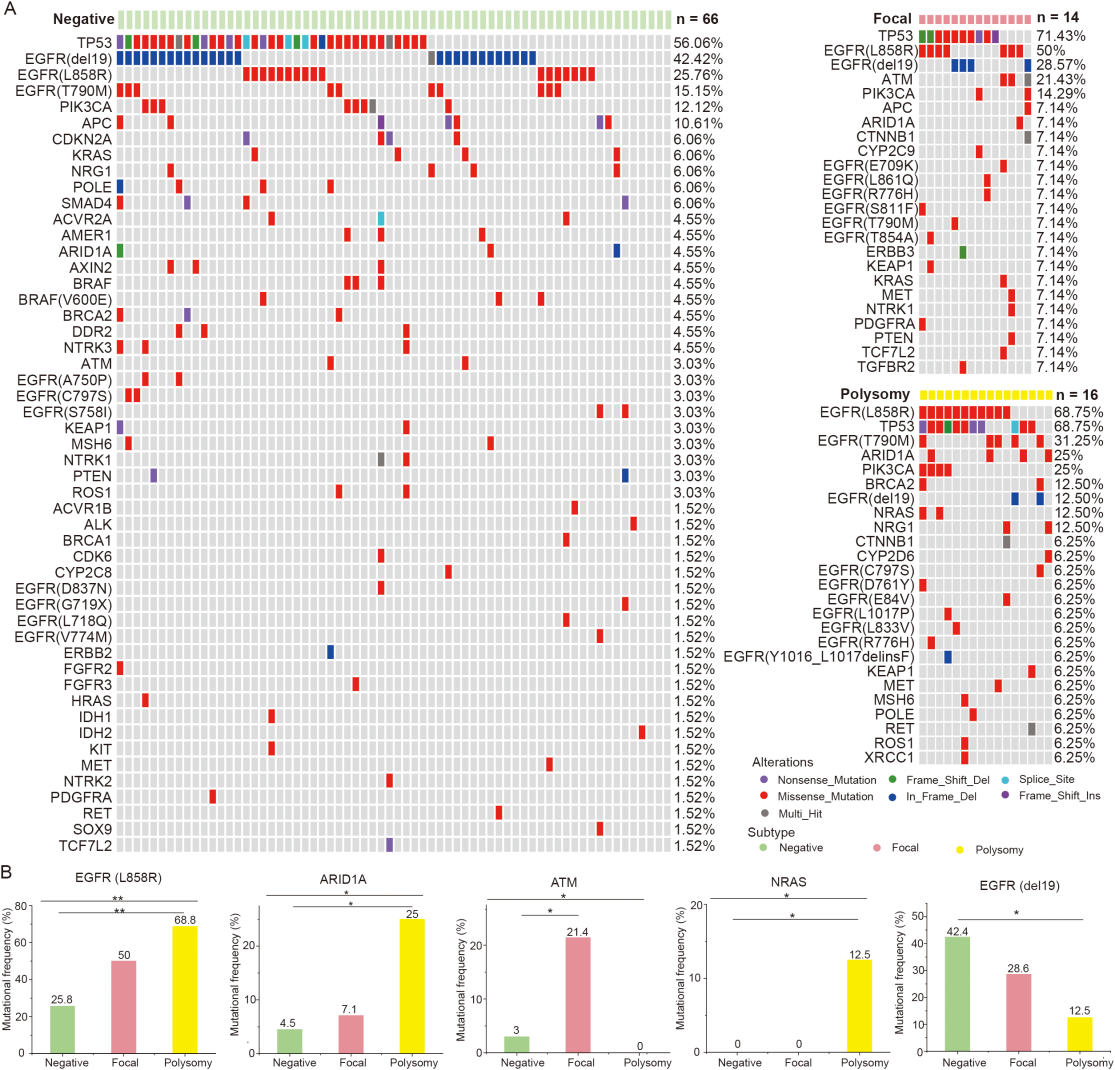
Correlations between genetic mutations and *MET* amplification status. **(A)** The gene mutational landscapes of NSCLC patients with *MET* amplification, polysomy and *MET* negative. **(B)** Genes with significantly different mutation frequencies in *MET* amplification, polysomy and *MET* negative groups. Amp, amplification. * represents *p* < 0.05 and ** represents *p* < 0.01.

Then, we displayed the mutation landscapes of NSCLC patients with *MET* amplification, polysomy and *MET* negative, respectively (**Figure 4A**), and explored the correlations between genetic mutations and *MET* amplification status. The results showed that frequencies of mutations such as *EGFR* L858R (50.0% vs. 68.8% vs. 25.8%, *p* = 0.004), *ARID1A* (7.1% vs. 25.0% vs. 4.5%, *p* = 0.033), *ATM* (21.4% vs. 0% vs. 3.0%, *p* = 0.045), *NARS* (0% vs. 12.5% vs. 0%, *p* = 0.046) were differed among *MET* amplification, polysomy and *MET* negative groups. After multiple comparisons, we found that NSCLC patients with polysomy had a significantly higher incidence of *EGFR* L858R (*p* = 0.002), *ARID1A* (*p* = 0.025) and *NRAS* (*p* = 0.036) and significantly lower incidence of *EGFR* del19 (*p* = 0.041) mutation compared with those with *MET* negative. Moreover, mutation in *ATM* (*p* = 0.035) was significantly more common in NSCLC patients with *MET* amplification compared with those with *MET* negative. In addition, no association was found between other mutations and *MET* amplification status (**Figure 4B**). These findings suggested that there may be a potential link between *MET* amplification status and gene mutations.

### 3.5 The known osimertinib resistance mechanisms disclosed by NGS in our cohort

**Figure 5:**
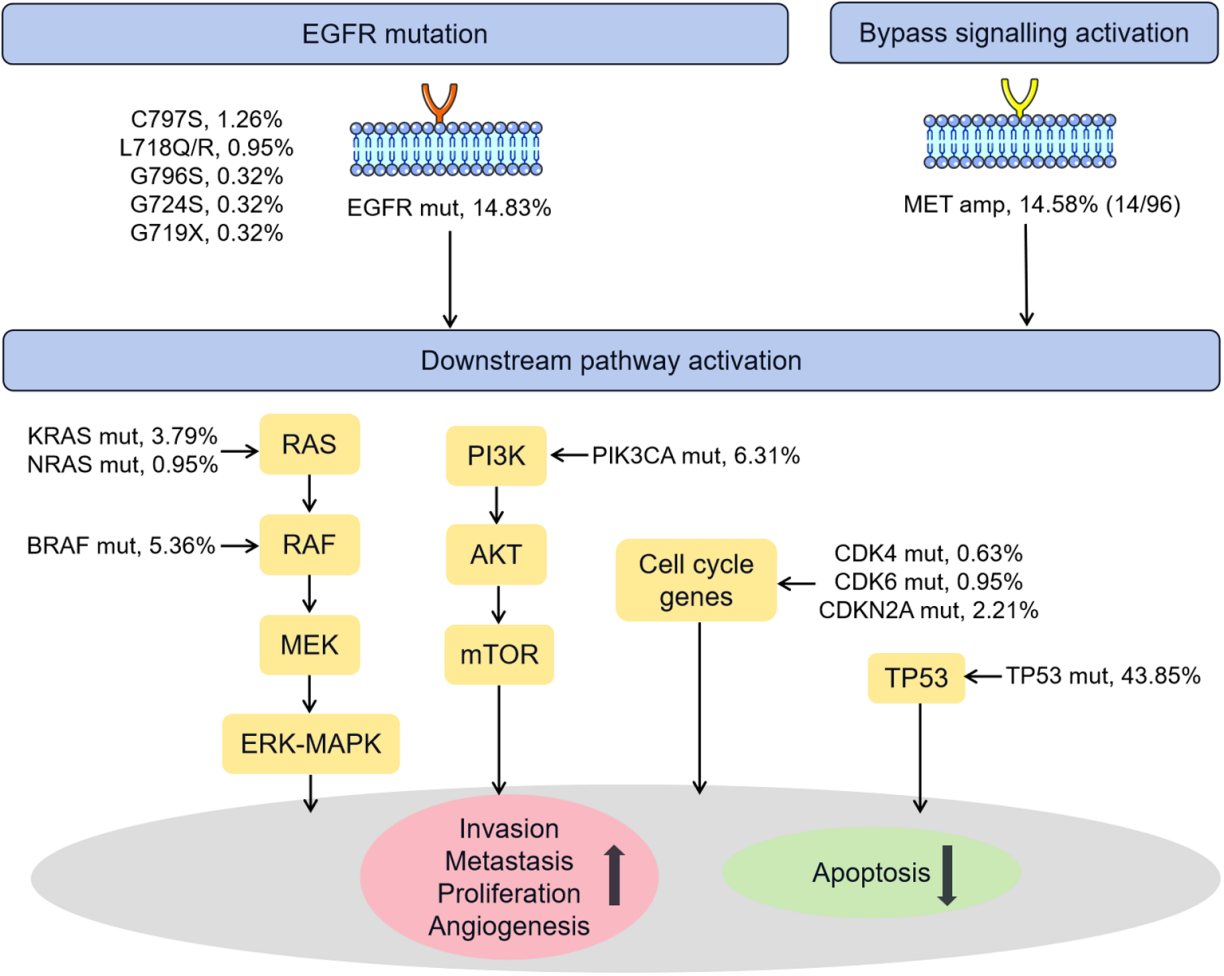
Schematic representation of the known mechanisms of resistance to osimertinib identified in our cohort. Amp, amplification; Mut, mutation.

**Figure 5** summarized the known genetic alterations associated with osimertinib resistance identified in our study. It is well recognized that the osimertinib resistance mechanisms are broadly divided into *EGFR*-dependent (on-target) and *EGFR*-independent (off-target) mechanisms. In our cohort, the known on-target mutations in C797S (1.26%), L718Q/R (0.95%), G796S (0.32%), G724S (0.32%), G719X (0.32%) sites were identified in several patients (33). Regarding *EGFR*-independent mechanisms, the known off-target alterations, such as *MET* amplification (14 of 96, 14.58%, bypass signaling activation), *TP53* mutation (43.85%) (34, 35), *PIK3CA* mutation (6.31%, PI3K/AKT/PTEN/mTOR pathway activation) (33), *BRAF*/*KRAS*/*NRAS* mutation (5.36%/3.79%/0.95%, RAS-RAF-MEK-MAPK pathway activation) (36), and *CDK4*/*CDK6*/*CDKN2A* mutation (0.63%/0.95%/2.21%, cell cycle gene) (37, 38), were observed in 52.05% of the current cohort.

## 4 Discussion

The use of reliable methods for the detection of *MET* amplification status in NSCLC is essential for identifying patients eligible for treatment with *MET* inhibitors. In the present study, the *MET* amplification status identified by the optimal model demonstrated excellent agreement with the FISH results, suggesting that NGS may be an alternative method for *MET* amplification detection. In addition, our study reported the comprehensive mutation profile of a largest cohort of Chinese NSCLC patients with osimertinib resistance, which may contribute to the discovery of potential resistance mechanisms and the development of new *EGFR*-TKIs.

In recent years, NGS has been widely applied in clinical practice for the detection of comprehensive gene profiles, including gene mutations, gene amplification, rearrangement, and fusion. However, it is still lacking robust evidence regarding the feasibility and appropriateness of NGS as an alternative method to FISH to identify *MET* amplification. To date, several studies have investigated the performance of NGS in *MET* amplification detection using FISH as the gold standard (16, 39). Unfortunately, the concordance between the FISH and NGS in identifying *MET* amplification status was low. For example, Lai et al. (21) reported the low correlation of *MET* amplification results obtained by NGS and FISH, where of the 18 NSCLC patients identified as FISH-positive (2 *MET* amplification and 16 polysomy), only 8 (44.4%) were deemed to have *MET* GCN gain according to NGS. Also, the TATTON study reported that among 47 FISH-positive patients, only 12 (27%) were diagnosed with *MET* amplification by NGS (40). In addition, the concordance rate among NGS and FISH was only 62.5% (25 of 40) in the study performed by Peng et al. (22). In our study, the optimal model in identifying *MET* amplification status achieved a relatively high concordance rate of 74.0% with FISH for detecting *MET* amplification status.

Notably, accumulated evidences have suggested that the *MET* amplification is the truly oncogenic driver of lung cancer. NSCLC patients with *MET* amplification presented a more robust response to *MET* inhibitors compared with those with polysomy in the published results of clinical trials (41, 42). Our study prioritized the identification of *MET* amplification. In terms of the identification of *MET* amplification, our NGS method achieved a sensitivity of 85.7% with FISH, providing a reliable measurement of this biomarker in the clinic. In contrast to previous studies, which only used single gene as the determinant to distinguish *MET* amplification from polysomy (16, 21), we utilized *CYP3A4*, *CDK6*, and *BRAF* genes to determine *MET* amplification, which allowed for its potential higher resolution in the discrimination of *MET* amplification status. Moreover, previous studies indicated that high-level amplified *MET* has been used as a biomarker to predict the benefit of *MET* inhibitors, emphasizing the importance of identifying high levels of *MET* amplification (43, 44). In our study, a 100% consistency rate between NGS and FISH was observed in samples harboring *MET* GCN ≥ 5, which indicated that high-level amplified *MET* samples can be reliably detected via NGS. This finding was consistent with the previous study performed by Schubart et al. (43), who reported that the NGS method showed the best concordance with FISH when *MET* GCN was > 10 (80%, 4 of 5). Interestingly, Xiang et al. (41) found that IHC (H-score ≥ 200) showed high overall consistency with FISH in identifying *MET* amplification. Hartmaier et al. (45) reported that 81.25% (13 of 16) cases of *MET* amplification by FISH were also IHC positive, and IHC expression tended to increase with increasing *MET* GCN (≥10) in FISH-positive tumors. These studies indicated the potential relationship between *MET* amplification by FISH and *MET* IHC. It would be of interest to look at the correlation of *MET* amplification by NGS with *MET* IHC in the future.

Over the past few decades, genetic alterations of cancer driver genes have been identified in NSCLC, and molecular testing and targeted therapies have become standard care for NSCLC patients (46). Osimertinib is currently the preferred first-line therapy in patients with NSCLC with common *EGFR* mutation and the standard second-line therapy in T790M-positive patients in progression to previous *EGFR*-TKIs (47, 48). Osimertinib is a highly effective treatment with a high response rate and long-lasting disease control. However, the resistance to the treatment inevitably develops among patients (38, 49). Therefore, a comprehensive understanding of genetic alterations in osimertinib resistant patients is crucial to characterize emerging molecular resistance mechanism and develop novel targeted treatment. Our study presented comprehensive mutation profile of a largest cohort of Chinese NSCLC patients with osimertinib resistance, which may provide some clinically valuable clues to understand the mechanism of osimertinib resistance. To date, a number of studies have revealed that resistance mechanisms to osimertinib are highly complex, including *EGFR*-dependent and *EGFR*-independent mechanisms (8, 48, 50). The *EGFR*-dependent mechanisms include *EGFR* mutations or amplifications, where the most common *EGFR* mutation is *EGFR* C797S, accounts for 0-29% of cases of resistance to osimertinib (48). In our study, the incidence of C797S mutations was 1.26%. Besides C797S mutations, several other known on-target mutations in L718Q/R (0.95%), G796S (0.32%), G724S (0.32%), G719X (0.32%) were also identified. Regarding *EGFR*-independent mechanisms, it has been reported that osimertinib resistance can be acquired by bypass pathway activation, downstream pathway activation, cell cycle gene mutation, and histologic transformation (33, 35, 36, 51). In our study, the off-target alterations were dominant, mainly in *TP53* mutation and *MET* amplification. It is worth noting that besides the discovery of the known genetic alterations medicating osimertinib resistance, a number of on-target mutations such as *EGFR* A750P (1.58%), E709K (0.63%), L861Q (0.63%), R776 (0.63%), S752F (0.63%), S758I (0.63%), and V536M as well as off-target mutations such as *NRG1* (9.46%), *ATM* (5.36%), *APC* (4.42%), *ARID1A* (4.42%), *NTRK1* (4.10%), *POLE* (4.10%), were also identified in multiple patients in our cohort. This finding may provide important guidance for future oncology efforts, such as exploring the potential resistance mechanisms and developing new *EGFR*-TKIs or combined strategies for NSCLC patients.

Several limitations of this study should be stated. First, regarding the comparison of FISH and NGS results, this study involved a relatively small sample size and lacked a validation cohort, which may affect the generalizability of our NGS methods for identifying *MET* amplification status. Second, no treatment response and prognosis data were available in this study. Further research is needed to validate the clinical utility of our NGS methods in determining tumors with *MET* amplification. We intend to carry out a larger sample size study to further validate our finding and examine the clinical utility of *MET* amplification by our NGS methods.

Collectively, this study demonstrated the potential of NGS as an alternative method in identifying *MET* amplification. In addition to *MET* amplification, DNA-based NGS assay could provide other data, such as characterizing various genetic alterations, which may potentially serve as an effective tool for guiding therapeutic strategies.

## Data Availability

The datasets used and/or analyzed during the current study are available from the corresponding author on reasonable request.

## References

[1] Sadeghirad H Bahrami T Layeghi SM Yousefi H Rezaei M Hosseini-Fard SR. Immunotherapeutic targets in non-small cell lung cancer. Immunology. (2023) 168:256–72. doi: 10.1111/imm.v168.2 35933597

[2] Rad HS Rad HS Shiravand Y Radfar P Arpon D Warkiani ME. The Pandora's box of novel technologies that may revolutionize lung cancer. Lung Cancer. (2021) 159:34–41. doi: 10.1016/j.lungcan.2021.06.022 34304051

[3] Wang Q Yang S Wang K Sun SY. MET inhibitors for targeted therapy of EGFR TKI-resistant lung cancer. J Hematol Oncol. (2019) 12:63. doi: 10.1186/s13045-019-0759-9 31227004 PMC6588884

[4] Remon J Steuer CE Ramalingam SS Felip E. Osimertinib and other third-generation EGFR TKI in EGFR-mutant NSCLC patients. Ann Oncol. (2018) 29:i20–7. doi: 10.1093/annonc/mdx704 29462255

[5] Lee DH. Treatments for EGFR-mutant non-small cell lung cancer (NSCLC): The road to a success, paved with failures. Pharmacol Ther. (2017) 174:1–21. doi: 10.1016/j.pharmthera.2017.02.001 28167215

[6] Wu SG Shih JY. Management of acquired resistance to EGFR TKI-targeted therapy in advanced non-small cell lung cancer. Mol Cancer. (2018) 17:38. doi: 10.1186/s12943-018-0777-1 29455650 PMC5817870

[7] Russo A Franchina T Ricciardi GRR Smiroldo V Picciotto M Zanghì M. Third generation EGFR TKIs in EGFR-mutated NSCLC: Where are we now and where are we going. Crit Rev Oncol Hematol. (2017) 117:38–47. doi: 10.1016/j.critrevonc.2017.07.003 28807234

[8] Leonetti A Sharma S Minari R Perego P Giovannetti E Tiseo M. Resistance mechanisms to osimertinib in EGFR-mutated non-small cell lung cancer. Br J Cancer. (2019) 121:725–37. doi: 10.1038/s41416-019-0573-8 PMC6889286 31564718

[9] Gomatou G Syrigos N Kotteas E. Osimertinib resistance: molecular mechanisms and emerging treatment options. Cancers (Basel). (2023) 15:841. doi: 10.3390/cancers15030841 36765799 PMC9913144

[10] Lutterbach B Zeng Q Davis LJ Hatch H Hang G Kohl NE. Lung cancer cell lines harboring MET gene amplification are dependent on Met for growth and survival. Cancer Res. (2007) 67:2081–8. doi: 10.1158/0008-5472.CAN-06-3495 17332337

[11] Robinson KW Sandler AB. The role of MET receptor tyrosine kinase in non-small cell lung cancer and clinical development of targeted anti-MET agents. Oncologist. (2013) 18:115–22. doi: 10.1634/theoncologist.2012-0262 PMC3579594 23345546

[12] Lee M Jain P Wang F Ma PC Borczuk A Halmos B. MEt alterations and their impact on the future of non-small cell lung cancer (NSCLC) targeted therapies. Expert Opin Ther Targets. (2021) 25:249–68. doi: 10.1080/14728222.2021.1925648 33945380

[13] Yin W Cheng J Tang Z Toruner G Hu S Guo M. MET amplification (MET/CEP7 ratio ≥ 1.8) is an independent poor prognostic marker in patients with treatment-naive non-small-cell lung cancer. Clin Lung Cancer. (2021) 22:e512–8. doi: 10.1016/j.cllc.2020.11.002 33288441

[14] Schildhaus HU Schultheis AM Rüschoff J Binot E Merkelbach-Bruse S Fassunke J. MET amplification status in therapy-naïve adeno- and squamous cell carcinomas of the lung. Clin Cancer Res. (2015) 21:907–15. doi: 10.1158/1078-0432.CCR-14-0450 25492085

[15] Westover D Zugazagoitia J Cho BC Lovly CM Paz-Ares L. Mechanisms of acquired resistance to first- and second-generation EGFR tyrosine kinase inhibitors. Ann Oncol. (2018) 29:i10–9. doi: 10.1093/annonc/mdx703 PMC6454547 29462254

[16] Qin K Hong L Zhang J Le X. MET amplification as a resistance driver to TKI therapies in lung cancer: clinical challenges and opportunities. Cancers (Basel). (2023) 15:612. doi: 10.3390/cancers15030612 36765572 PMC9913224

[17] Remon J Hendriks LEL Mountzios G García-Campelo R Saw SPL Uprety D. MEt alterations in NSCLC-current perspectives and future challenges. J Thorac Oncol. (2023) 18:419–35. doi: 10.1016/j.jtho.2022.10.015 36441095

[18] Garon EB Brodrick P. Targeted therapy approaches for MET abnormalities in non-small cell lung cancer. Drugs. (2021) 81:547–54. doi: 10.1007/s40265-021-01477-2 33638808

[19] Duncan DJ Vandenberghe ME Scott MLJ Barker C. Fast fluorescence in situ hybridisation for the enhanced detection of MET in non-small cell lung cancer. PLoS One. (2019) 14:e0223926. doi: 10.1371/journal.pone.0223926 31613934 PMC6793848

[20] Clavé S Jackson JB Salido M Kames J Gerding KMR Verner EL. Comprehensive NGS profiling to enable detection of ALK gene rearrangements and MET amplifications in non-small cell lung cancer. Front Oncol. (2023) 13:1225646. doi: 10.3389/fonc.2023.1225646 37927472 PMC10623306

[21] Lai GGY Lim TH Lim J Liew PJR Kwang XL Nahar R. Clonal MET amplification as a determinant of tyrosine kinase inhibitor resistance in epidermal growth factor receptor-mutant non-small-cell lung cancer. J Clin Oncol. (2019) 37:876–84. doi: 10.1200/JCO.18.00177 30676858

[22] Peng LX Jie GL Li AN Liu SY Sun H Zheng MM. MET amplification identified by next-generation sequencing and its clinical relevance for MET inhibitors. Exp Hematol Oncol. (2021) 10:52. doi: 10.1186/s40164-021-00245-y 34758872 PMC8579577

[23] Sun B Qiu T Zeng X Duan J Bai H Xu J. Detection of MET polysomy by next-generation sequencing and its clinical relevance for MET inhibitors. Cancer Res Commun. (2023) 3:532–9. doi: 10.1158/2767-9764.CRC-22-0438 PMC10072163 37025355

[24] Kumaki Y Olsen S Suenaga M Nakagawa T Uetake H Ikeda S. Comprehensive genomic profiling of circulating cell-free DNA distinguishes focal MET amplification from aneuploidy in diverse advanced cancers. Curr Oncol. (2021) 28:3717–28. doi: 10.3390/curroncol28050317 PMC8534719 34677235

[25] Bai Q Shi X Zhou X Liang Z Lu S Wu Y. Chinese expert consensus on clinical practice of MET detection in non-small cell lung cancer. Zhonghua Bing Li Xue Za Zhi. (2022) 51:1094–103. doi: 10.1177/17588359231216096 36323537

[26] Cibulskis K Lawrence MS Carter SL Sivachenko A Jaffe D Sougnez C. Sensitive detection of somatic point mutations in impure and heterogeneous cancer samples. Nat Biotechnol. (2013) 31:213–9. doi: 10.1038/nbt.2514 PMC3833702 23396013

[27] Boutros PC Ewing AD Ellrott K Norman TC Dang KK Hu Y. Global optimization of somatic variant identification in cancer genomes with a global community challenge. Nat Genet. (2014) 46:318–9. doi: 10.1038/ng.2932 PMC4035501 24675517

[28] Wang K Li M Hakonarson H. ANNOVAR: functional annotation of genetic variants from high-throughput sequencing data. Nucleic Acids Res. (2010) 38:e164. doi: 10.1093/nar/gkq603 20601685 PMC2938201

[29] Lek M Karczewski KJ Minikel EV Samocha KE Banks E Fennell T. Analysis of protein-coding genetic variation in 60,706 humans. Nature. (2016) 536:285–91. doi: 10.1038/nature19057 PMC5018207 27535533

[30] Abecasis GR Auton A Brooks LD DePristo MA Durbin RM Handsaker RE. An integrated map of genetic variation from 1,092 human genomes. Nature. (2012) 491:56–65. doi: 10.1038/nature11632 23128226 PMC3498066

[31] Kumar P Henikoff S Ng PC. Predicting the effects of coding non-synonymous variants on protein function using the SIFT algorithm. Nat Protoc. (2009) 4:1073–81. doi: 10.1038/nprot.2009.86 19561590

[32] Adzhubei IA Schmidt S Peshkin L Ramensky VE Gerasimova A Bork P. A method and server for predicting damaging missense mutations. Nat Methods. (2010) 7:248–9. doi: 10.1038/nmeth0410-248 PMC2855889 20354512

[33] Zalaquett Z Catherine Rita Hachem M Kassis Y Hachem S Eid R Raphael Kourie H. Acquired resistance mechanisms to osimertinib: The constant battle. Cancer Treat Rev. (2023) 116:102557. doi: 10.1016/j.ctrv.2023.102557 37060646

[34] Vokes NI Chambers E Nguyen T Coolidge A Lydon CA Le X. Concurrent TP53 mutations facilitate resistance evolution in EGFR-mutant lung adenocarcinoma. J Thorac Oncol. (2022) 17:779–92. doi: 10.1016/j.jtho.2022.02.011 PMC10478031 35331964

[35] Ibusuki R Iwama E Shimauchi A Tsutsumi H Yoneshima Y Tanaka K. TP53 gain-of-function mutations promote osimertinib resistance via TNF-α-NF-κB signaling in EGFR-mutated lung cancer. NPJ Precis Oncol. (2024) 8:60. doi: 10.1038/s41698-024-00557-2 38431700 PMC10908812

[36] He J Huang Z Han L Gong Y Xie C. Mechanisms and management of 3rd−generation EGFR−TKI resistance in advanced non−small cell lung cancer (Review). Int J Oncol. (2021) 59:90. doi: 10.3892/ijo.2021.5270 34558640 PMC8562388

[37] Volta F La Monica S Leonetti A Gnetti L Bonelli M Cavazzoni A. Intrinsic resistance to osimertinib in EGFR mutated NSCLC cell lines induced by alteration in cell-cycle regulators. Target Oncol. (2023) 18:953–64. doi: 10.1007/s11523-023-01005-0 PMC10663255 37855989

[38] Bertoli E De Carlo E Del Conte A Stanzione B Revelant A Fassetta K. Acquired resistance to osimertinib in EGFR-mutated non-small cell lung cancer: how do we overcome it? Int J Mol Sci. (2022) 23:6936. doi: 10.3390/ijms23136936 35805940 PMC9266773

[39] Heydt C Becher AK Wagener-Ryczek S Ball M Schultheis AM Schallenberg S. Comparison of in situ and extraction-based methods for the detection of MET amplifications in solid tumors. Comput Struct Biotechnol J. (2019) 17:1339–47. doi: 10.1016/j.csbj.2019.09.003 PMC6861603 31762957

[40] Hartmaier RJ Markovets AA Ahn MJ Sequist LV Han JY Cho BC. Osimertinib + Savolitinib to overcome acquired MET-mediated resistance in epidermal growth factor receptor-mutated, MET-amplified non-small cell lung cancer: TATTON. Cancer Discovery. (2023) 13:98–113. doi: 10.1158/2159-8290.CD-22-0586 36264123 PMC9827108

[41] Xiang C Lv X Chen K Guo L Zhao R Teng H. Unraveling the significance of MET focal amplification in lung cancer: integrative NGS, FISH, and IHC investigation. Mod Pathol. (2024) 37:100451. doi: 10.1016/j.modpat.2024.100451 38369190

[42] Solomon JP Yang SR Choudhury NJ Ptashkin RN Eslamdoost N Falcon CJ. Bioinformatically expanded next-generation sequencing analysis optimizes identification of therapeutically relevant MET copy number alterations in >50,000 tumors. Clin Cancer Res. (2022) 28:4649–59. doi: 10.1158/1078-0432.CCR-22-1321 PMC9633455 36044468

[43] Schubart C Stöhr R Tögel L Fuchs F Sirbu H Seitz G. MET amplification in non-small cell lung cancer (NSCLC)-A consecutive evaluation using next-generation sequencing (NGS) in a real-world setting. Cancers (Basel). (2021) 13:5023. doi: 10.3390/cancers13195023 34638507 PMC8508248

[44] Camidge DR Otterson GA Clark JW Ignatius Ou SH Weiss J Ades S. Crizotinib in patients with MET-amplified NSCLC. J Thorac Oncol. (2021) 16:1017–29. doi: 10.1016/j.jtho.2021.02.010 33676017

[45] Hartmaier R Han JY Cho BC Markovets A Kurian N Cantarini M. Abstract CT127: Tumor response and MET-detection methods exploratory biomarker analysis of Part B of the Ph 1b TATTON study. Cancer Res. (2021) 81 (13_Supplement):CT127. doi: 10.1158/1538-7445.AM2021-CT127

[46] Du Z Zhang Y Hesilaiti N Xia Q Cui H. Structure-guided strategies of targeted therapies for patients with EGFR-mutant non-small cell lung cancer. Biomolecules. (2023) 13:210. doi: 10.3390/biom13020210 36830579 PMC9953181

[47] Tang ZH Lu JJ. Osimertinib resistance in non-small cell lung cancer: Mechanisms and therapeutic strategies. Cancer Lett. (2018) 420:242–6. doi: 10.1016/j.canlet.2018.02.004 29425688

[48] Fu K Xie F Wang F Fu L. Therapeutic strategies for EGFR-mutated non-small cell lung cancer patients with osimertinib resistance. J Hematol Oncol. (2022) 15:173. doi: 10.1186/s13045-022-01391-4 36482474 PMC9733018

[49] Yang Z Yang N Ou Q Xiang Y Jiang T Wu X. Investigating novel resistance mechanisms to third-generation EGFR tyrosine kinase inhibitor osimertinib in non-small cell lung cancer patients. Clin Cancer Res. (2018) 24:3097–107. doi: 10.1158/1078-0432.CCR-17-2310 29506987

[50] Dong RF Zhu ML Liu MM Xu YT Yuan LL Bian J. EGFR mutation mediates resistance to EGFR tyrosine kinase inhibitors in NSCLC: From molecular mechanisms to clinical research. Pharmacol Res. (2021) 167:105583. doi: 10.1016/j.phrs.2021.105583 33775864

[51] Roper N Brown AL Wei JS Pack S Trindade C Kim C. Clonal evolution and heterogeneity of osimertinib acquired resistance mechanisms in EGFR mutant lung cancer. Cell Rep Med. (2020) 1:100007. doi: 10.1016/j.xcrm.2020.100007 32483558 PMC7263628

